# 

**Published:** 1874-09

**Authors:** 


### TABLE OF CONTENTS.

ORIGINAL COMMUNICATIONS.

The Rattlesnake—Its Poison and Antidote. By G. M. Rivers, M. D., S. C. - 505

Atropia in Collapse. By T. Curtis Smith, M. D., of Middleport, Ohio. - 513

Medicine in its Relations with Botany—A Chair of Botany in Every Medical School—No. 3. By W. T. Grant, M.D., of Georgia - - - - 517

The Mind Kills the Body— Extract. By J. C. Berry, M.D., West Virginia. 522

Fracture of the Clavicle. By A. R. Kilpatrick, M.D., of Texas. - - 523

REMARKS, GLEANINGS AND EXTRACTS.

Mercury in Syphilis—525. A Singular Case—526. Abdominal Wounds—527. Treatment of Whooping-Cough by Quinine—528. The Effect of Alcohol on Digestion; Drunkenness and Insanity—529. A Case of Camphor-Poisoning—530. Extraction of a Foreign Body from the Male Urethra; Retro-Peritoneal Hernia—531. Ergot as an Hæmostatic; Obstinate Vomiting of Pregnancy Cured by Enemata of Bromide of Potassium; Union of Two Kidneys in One—532. Ergotine Injection in Prolapsus Ani; A Novel Remedy for Whooping-Cough; The Use of Chloral Hydrate—533. Ergot in Hemorrhage; Coryza—534. Potato Bugs as Good as Spanish Flies; Chronic Diarrhœa in Infants and Children; The Respiratory Centre; The Production of Anæsthesia During Sleep—535. Chlorhydrate of Trimethylamin in Rheumatic Fever; Enemata of Bromide of Potassium in Obstinate Vomiting; Lemon-juice in Diphtheria—536. Hypodermic Injection of Ergot in Varicocele; Action of Bromide of Camphor; Acetic Acid Spray in Diphtheria; Surgical Anæsthesia—537. "Cincho-Quinine"—538. Topical Remedies in Diseases of the Throat, Nose and Ear—539. Treatment of Angina Dipththeritica—540. Ipecac in Delirium Tremens; Treatment of Ascarides—541. McIntosh on Dysmenorrhœa and its Treatment with Sulphate of Quinia and Extract of Stramonium Seeds; Invalid Diet and Cookery—542. Bismuth as an External Application in Skin Diseases; Podophyllin—543. Digitalis in Dropsy; Lobelia—544. The Treatment of Syphilis; Pleurisy—545. Phosphorus in Neuralgia; Treatment of the Acute Stage of Blenorrhagia with Cannabis Indica and Benzoic Acid; Treatment of Ulcerations of the Neck of the Uterus—546. Tincture of Veratrum in Pneumonia in Children; Rhus Toxicodendron in Erysipelas—547. Treatment of Sloughy Ulcers of the Vulva; Chloral Suppositories—548. Best Method of Using Chlorate of Potash in Mercurial Stomatitis; Chloral Hydrate in Insomnia of Infants; External Use of Chlorate of Potash in Open Cancers; Aneurism of the Arch of the Aorta—549. Incontinence of Urine in Children; Treatment of Constipation; Treatment of Salivation by Atropia; Solution of Camphor in Erysipelas—550. Treatment of Sore Nipples; Successful Employment of Propylamin in Acute Articular Rheumatism; Use of Pepsin in the Treatment of Dyphtheria—551. Powdered Coal Tar for Wounds; Sulphate of Cinchonidia in Intermittent Fever; Cure for the Itch—552. Perpetual Paste; Treatment of Shingles; Uses of Chloral; Formula for the Troublesome Cough of Phthisis; For Constipation—553. To Keep Away Flies; Bromide of Ammonium in Catamenial Excesses; Cannabis Indica in the Treatment of Migraine; The Value of Potatoes; How to Remove Adhesive Plasters; Pancoat's Styptic—554. A Simple Method of Reducing the Dislocation of the Forearm Backwards; Treatment of Paraphymosis; Pathognomonic Sign of Pertussis; Hæmorrhoids; Agreeable Purgative; Formula for Tasteless Cod-Liver Oil.—555. New Mode of Treating Conjunctivitis; Iodid Acid in Tumors; Silicate of Soda in Gonorrhœa; Nocturnal Incontinence of Urine; Treatment of Burns; Formula for Syrup of Pepsin—556. Stricture of Cervix and Perineum; For Gastralgia; Popular Antacid Soda Mint; Freckles; Formula for the Relief of Flatus—557. The Apothysal Point in Neuralgia; Ulceration of Rectum; Treatment; Hydrastin; Scorbutus; Pomatum for the Prevention and Cure of Baldness—558. Retention of Urine; Chancroids; Mode of Administering Creasote; Unrethrotomy in the Aged—559.

EDITORIAL AND MISCELLANEOUS.

To Our Subscribers. - . - - - - - 560

Dr. D. E. S. - - - - - - - - - 561

Books and Pamphlets - - - - - - - - - 563

